# Synthesis of Fe-MCM-41 Using Iron Ore Tailings as the Silicon and Iron Source

**DOI:** 10.1155/2012/928720

**Published:** 2012-04-03

**Authors:** Xin Li, Honghao Yu, Yan He, Xiangxin Xue

**Affiliations:** ^1^School of Material Science and Engineering, Shenyang Ligong University, Shenyang 110159, China; ^2^School of Material and Metalurgy, Northeastern University, Shenyang 110004, China

## Abstract

Highly ordered Fe-MCM-41 molecular sieve was successfully synthesized by using *n*-hexadecyl-trimethyl ammonium bromide (CTAB) as the template and the iron ore tailings (IOTs) as the silicon and iron source. X-ray diffraction (XRD), high-resolution transmission electron microscopy (HRTEM), diffuse reflectance UV-visible spectroscopy, ^29^Si magic-angle spinning (MAS) nuclear magnetic resonance (NMR), and nitrogen adsorption/desorption were used to characterize the samples. The results showed that the mesoporous materials had highly ordered 2-dimensional hexagonal structure. The synthesized sample had high surface area, and part of iron atoms is retained in the framework with formation of tetrahedron after removal of the template by calcinations. The results obtained in the present work demonstrate the feasibility of employing iron ore tailings as a potential source of silicon and iron to produce Fe-MCM-41 mesoporous materials.

## 1. Introduction

MCM-41 materials are mesoporous silica with a hexagonal arrangement of pores and typical diameter of 1.5–10.0 nm [1]. Generally, pure silica MCM-41 has limited catalytic activity. Recently, several metal ions including Ti^4+^, Cr^6+^, Mo^5+^, V^5+^, and Fe^3+^ have been successfully incorporated into the frameworks of mesoporous silica [2–6]. Incorporation of transition metals into the framework of MCM-41 allows the preparation of catalysts, which are active in many different reactions such as oxidation of organic materials [7]. In particular, iron-containing mesoporous materials MCM-41 (Fe^3+^) have been extensively studied because of their unique catalytic enhancement of hydrocarbon oxidation, selective reduction, acylation, and alkylation reactions [8, 9]. Iron-containing mesoporous materials MCM-41 can be synthesized using a variety of silicon and iron precursors such as *n*-alkoxysilanes, *n*-alkylamines, sodium silicate, aerosil, ferric chloride, and ferric nitrate [10–13]. However, a drawback of these precursors is the high starting costs of the raw material that result in high production cost. Furthermore, the high toxicities of the preferred silica precursors and the structure directing agents used during synthesis require careful attention and handling facility to prevent pollution of the environment.

At present, the iron ore tailings (IOT) from the iron and steel industry are estimated to be more than 20 billion ton in China [14]. The problem with IOT lies in the fact that its disposal requires large quantities of land, and the utilization rate of IOT is rather low, approximately 7% of the generated amount [15, 16]. Most of IOTs are utilized in industry as a substitute for fine aggregates in cement and concrete, in bricks and ceramic tiles [17–19]. Owing to the high content of useful SiO_2_ and Fe_2_O_3_ (65–85 wt% and 10–20 wt%, resp.) in IOT, it makes perfect economic outlook to recover these minerals for industrial applications. IOT as starting material for the preparation of iron-containing mesoporous materials has not been explored in detail.

The novel synthesis of iron-containing mesoporous materials (Fe-MCM-41) material using IOT as new, cheap, and recycling silicon and iron sources is presented. The phase, textural properties, and morphology of synthesized Fe-MCM-41 were investigated. 

## 2. Materials and Methods

### 2.1. Starting Materials

In the present study, the production of Fe-MCM-41 involved the use of IOT as the silicon and iron source. IOTs were obtained from Anshan iron and steel group corporation in China. IOTs were initially ground by ball milling and dried in air at 373 K, and the mean particle size was determined at ca. 12 *μ*m by laser particle size analyzer [20]. The compositions of the IOTs were shown in Table 1. As listed in Table 1, IOTs were comprised of SiO_2_, Fe_2_O_3_, Al_2_O_3_, CaO, MnO, and TiO_2_.

The chemicals, such as tetraethoxysilane (TEOS 99.5%, Merck), sodium hydroxide (NaOH, 99.8%, Merck), *n*-hexadecyl-trimethyl ammonium bromide (CTAB, Merck), acetic acid (glacial, 100%, Merck), ammonia solution (25%, Merck) and ethanol (denatured, 99.5%, Merck), and ferric nitrate (Fe_2_(NO)_3_, 99.6%, Merck), were used in the as-received condition.

### 2.2. Extraction of Silicon and Iron Solution from IOT

Extraction of silicon and iron from IOT for the synthesis of Fe-MCM-41 was carried out under molten conditions without any addition of water. Molar composition of the mixtures was 1 IOT : 2.2 NaOH : 1 NaNO_3_. The mixtures were heated in molten state at 773 K for 3 h. After cooling to room temperature, the resultant lump was roughly crushed and ball milled for 24 h to prepare the mixtures of IOT powder. Then, the mixtures of IOT powder and water (solid : liquid = 1 : 5, volume ratio) in a 1000 mL sealed PP bottle were stirred at room temperature for 4.5 h. Finally, the solution was separated from the mixture by a filtration process. The amounts of silicon and iron in the extracted solution were 14.4 and 0.4 g L^−1^, respectively (analyzed by ICP-AES, Perkin-Elmer 3000 XL).

### 2.3. Synthesis of Fe-MCM-41

In a typical synthesis procedure, CTAB was dissolved in 60 mL of deionized water at room temperature. Then, the silicon and iron source from IOT and ferric nitrate were added to the CTAB solution. The pH was adjusted to 10.2 by using ammonia solution. The final molar composition of the gel was 0.15 CTAB : 1 Si : 0.02 Fe : 100 H_2_O. The mixture was stirred at room temperature for 1 h, placed in a teflon-lined autoclave, and heated at 373 K for 3 days. The resulting solid was filtered, washed repeatedly using deionized water, and air-dried overnight at 373 K. Finally, the as-synthesized Fe-MCM-41 powder was calcined at 823 K for 6 h to burn out the organic template to obtain surfactant-free mesoporous material.

### 2.4. Synthesis of MCM-41 from Pure Silica Source

Mesoporous materials prepared from pure silicon source were prepared for comparison purpose. In a typical synthesis procedure, the sample was obtained by the hydrolysis and cocondensation of TEOS at room temperature based on the method reported elsewhere by Matsumoto et al. [21]. The as-synthesized sample was calcined at 823 K for more than 6 h to burn out the template to obtain surfactant-free sample. 

### 2.5. Characterization

XRD was obtained with a Bruker NanoStar using filtered Cu *Kα* radiation. Diffraction data were recorded in the 2**θ** range of 1.5–8° and 10–80° at an interval of 0.02° with a scanning rate of 1° min^−1^. Nitrogen adsorption isotherms were measured by a Micromeritics ASAP 2020 system at 77 K. Prior to the measurement, samples were out-gassed at 153 K for at least 6 h. HRTEM images were obtained with a JEOL JEM-2200FS instrument operating at an accelerating voltage 200 kV. The samples were ultrasonically dispersed in ethanol and then dropped onto the carbon-coated copper grids prior to the measurements. ^29^Si-MAS NMR was recorded at 11.75T on Bruker MSL-300. Diffuse reflectance UV-visible spectroscopy measurements were recorded on a Shimadzu UV-2550 spectrometer fitted with an ISR-2200 integrating sphere attachment from 200 to 600 nm referenced to BaSO_4_.

## 3. Results and Discussion

### 3.1. XRD

**Figure 1:**
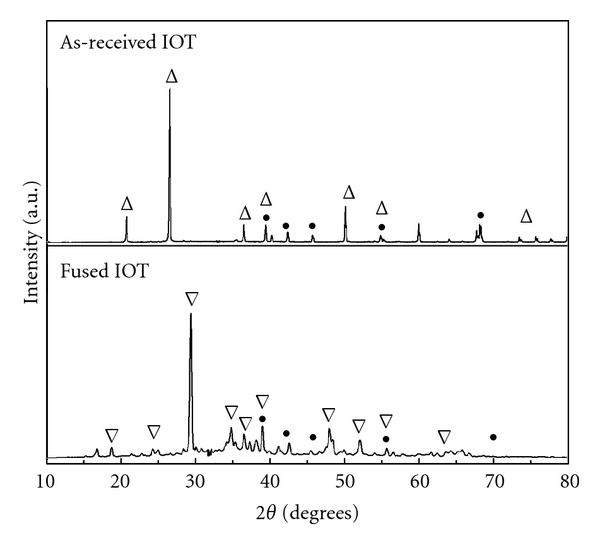
X-ray diffraction patterns of as-received IOT and fused IOT at 773 K. (Key: (∆) quartz, (*∇*) sodium silicate, and (●) hematite.)

The room temperature XRD patterns of as-received IOT and fused IOT at 773 K are shown in Figure 1. The XRD signatures of these powders revealed that the major crystalline phases found in the as-received IOT were quartz and hematite. In contrast, the XRD pattern of fused IOT exhibited major sodium silicate phase and minor hematite phase. The disappearance of quartz phases suggested that silica in its natural crystalline form had reacted with NaOH and NaNO_3_ to form soluble sodium silicate during the fusion process. Thus, it could be inferred that the fusion process employed in this work was effective in extracting silica from quartz to sodium silicate as a cheap and green silica source to produce Fe-MCM-41.

**Figure 2:**
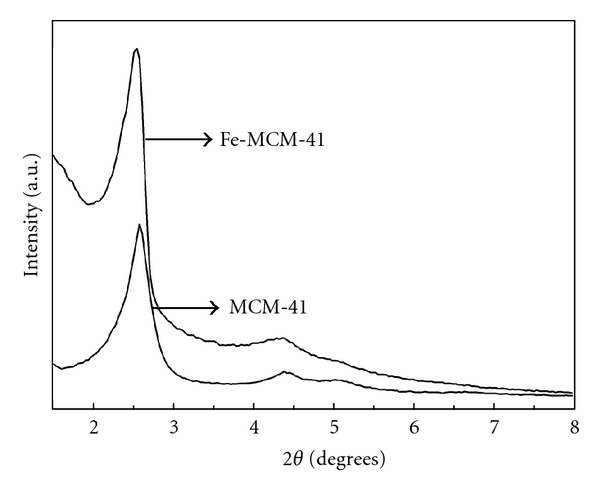
XRD patterns of MCM-41 and Fe-MCM-41.

XRD patterns of calcined Fe-MCM-41 and MCM-41 are shown in Figure 2. As shown in Figure 2, the XRD patterns of both Fe-MCM-41 and MCM-41 materials were in agreement with that reported by De Stefanis et al. [10] and Matsumoto et al. [21]. The XRD patterns of surfactant-free Fe-MCM-41 and MCM-41 exhibited one pronounce peak at 2.55° and 2.58°, respectively, that were attributed to the reflection from (100) plane. Two weak diffraction peaks at 2**θ**= 4.37° and 4.06° attributable to the reflection from (110) and (200) planes, respectively, were observed for Fe-MCM-41. In addition, two peaks at 2**θ**= 4.40° and 5.11° also attributable to the reflection from (110) and (200) planes, respectively, were observed for MCM-41. These results suggested that the successful formation of 2-dimensional hexagonal pore structure of typical MCM-41 materials and the pore structure were retained after removal of surfactant. 

The [100] peak shifted to a higher 2**θ**angle indicating a decrease in interplanar *d*-spacing from 3.46 nm to 3.42 nm for MCM-41 and Fe-MCM-41. This suggested that some Fe^3+^ ions had been incorporated in the framework and caused the expansion of the unit cell. In addition, the appearance of higher angle reflections indicated the existence of long-range order of 2-dimensional hexagonal structure as confirmed by TEM analysis.

### 3.2. BET

**Figure 3:**
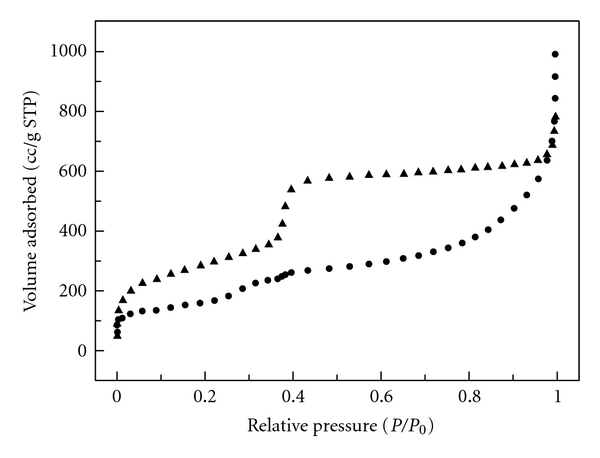
Nitrogen adsorption isotherms of MCM-41 and Fe-MCM-41.

The nitrogen adsorption isotherms of surfactant-free Fe-MCM-41 and MCM-41 are shown in Figure 3. Each isotherm was of type IVb in the IUPAC classifications, which was a typical isotherm for mesoporous materials [20, 22, 23]. The isotherms exhibited that the adsorption amount increased abruptly at relative pressure (*P*/*P*_0_) ca. 0.35–0.40 due to the capillary condensation in mesopores. The steep increase of the capillary condensation step suggested that the mesoporous materials had a rather uniform pore sizes. Moreover, the distinct abruption of the isotherms curve at a relative pressure of ca. 0.90 probably was attributed to interparticle pores in samples.

After sintered template, iron oxides from iron ions dispered in the pore wall surface. So the BET surface area of MCM-41 (ca. 1029 m^2^ g^−1^) was higher than that of Fe-MCM-41 (ca. 462 m^2^ g^−1^). Furthermore, this result indicated that mesoporous materials prepared from TEOS should be more reactive and presenting a larger amount of silicate anions, which would be present in the mixture. The higher amount of silicate anions could be condensed and hydrolyzated in a higher surface area [24]. 

### 3.3. UV-Visible

**Figure 4:**
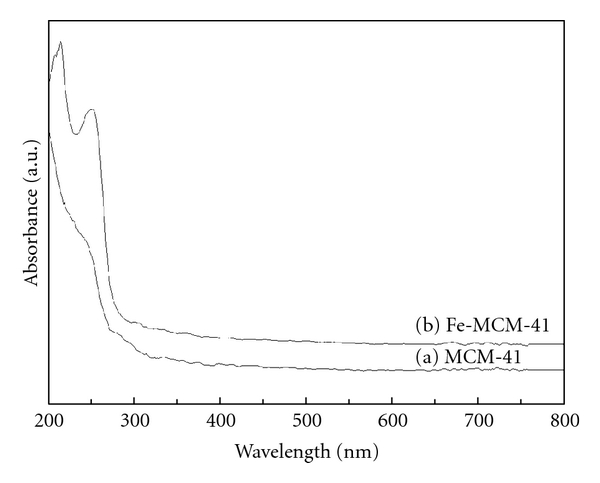
UV-visible spectra of MCM-41 and Fe-MCM-41.

Diffuse reflectance UV-visible spectroscopy was used to characterize the nature and coordination of Fe^3+^ [3] in the MCM-41 mesoporous molecular sieves.  Figure 4 shows UV-visible spectra of the calcined Fe-MCM-41 and MCM-41 as a function of metal loading. For MCM-41, there was not absorption bands in the 200–800 nm region. However, there were two prominent absorption bands in the UV-visible spectroscopy of calcined Fe-MCM-41 (Figure 4(b)). The strong absorption bands in the 200–300 nm region (two clearly distinguished peaks at *λ* = 214 and 250 nm) were attributed to the charge-transfer (CT) transitions involving isolated framework Fe^3+^ in (FeO)_4_^−^ tetrahedral geometry [25]. No formation of any absorption bands in the 200–300 nm region suggests that all the Fe^3+^ ions migrate in framework with formation of tetrahedral.

### 3.4. HRTEM

**Figure 5:**
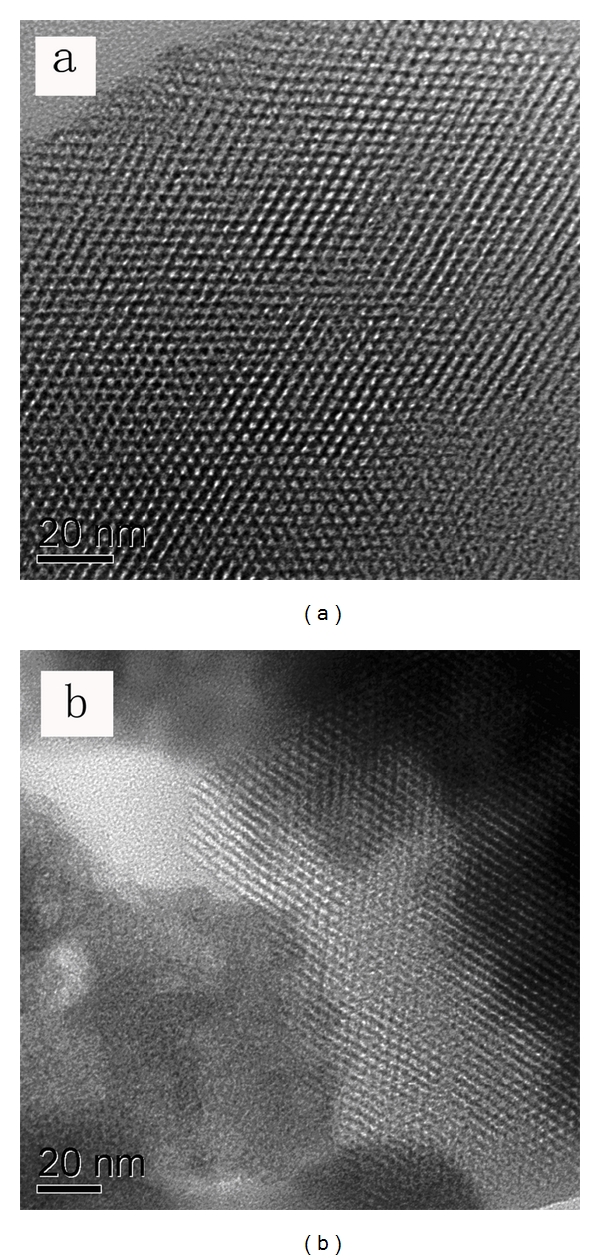
HRTEM images of (a) MCM-41 and (b) Fe-MCM-41.

A representative TEM micrograph of surfactant-free MCM-41 and Fe-MCM-41 taken along the [100] direction is shown in Figure 5. The mesoporous silica exhibited highly ordered hexagonal array of pore structure and streak structural features of typical MCM-41 materials. The diameter between the fringes of the pore (pore wall) and crystallinity of MCM-41 were greater than Fe-MCM-41. This suggested that some Fe^3+^ ions had been incorporated in the framework and caused the expansion of the unit cell, which is consistent with the results of low-angle XRD.

### 3.5. NMR

**Figure 6:**
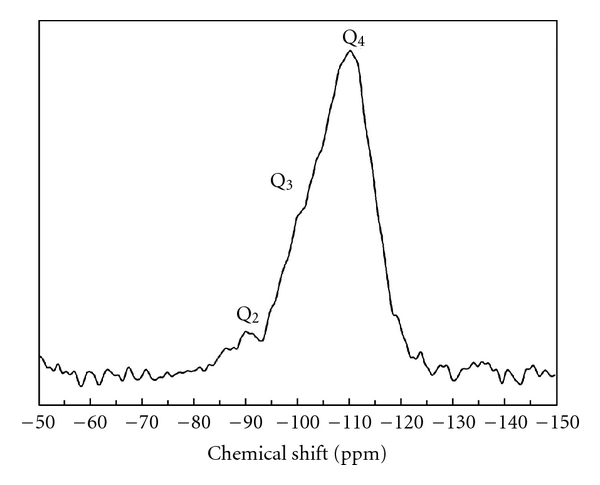
Solid-state ^29^Si NMR of calcined Fe-MCM-41.

The representative ^29^Si MAS NMR spectrum of surfactant-free Fe-MCM-41 is shown in Figure 6. The spectrum exhibited three resonance peaks at ca. −90 ppm, −100 ppm, and −110 ppm. The peak signals at −100 ppm and −110 ppm are attributed to the silicon atoms in Q^3^ [SiO_3_(OH)] of isolated silanols and Q^4^ [SiO_4_] environments of siloxane bridges, respectively. In addition, a small signal attributable to the silicon atoms in Q^2^ [SiO_2_(OH)_2_] environment of vicinal and geminal silanols was also observed.

## 4. Conclusions

Highly ordered Fe-MCM-41 molecular sieve was successfully synthesized by using the iron ore tailings as the silicon and iron source. The characterization of powder X-ray diffraction and high-resolution transmission electron microscope on Fe-MCM-41 material from the iron ore tailings indicated that the long-range order structure was achieved, and the regular mesoporous hexagonal structure of typical MCM-41 was obtained through surfactant removal. UV-visible spectra and nitrogen adsorption/desorption on Fe-MCM-41 material indicated the synthesized materials had high surface area and part of iron atom still retain in the framework with formation of tetrahedral after removal of the template by calcinations. This result showed that the iron ore tailings had the potential to be used as an alternative and cheap source of silica in the production of mesoporous Fe-MCM-41 materials.

## Figures and Tables

**Table 1:** Chemical composition of iron ore tailings (IOT).

Compound	SiO_2_	Fe_2_O_3_	Al_2_O_3_	CaO	MnO	TiO_2_
Content (wt%)	82.26	14.37	0.8	0.57	0.034	0.016
IOT (mol/100 g)	1.371	0.090	0.008	0.010	<0.001	<0.001
